# 성인초기 여성의 e헬스 문해력, 생식건강지식, 자아존중감이 건강증진행위에 미치는 영향: 설문조사연구

**DOI:** 10.4069/kjwhn.2022.12.15

**Published:** 2022-12-29

**Authors:** Hye Sook Shin, Young A Song

**Affiliations:** 1 College of Nursing Science, Kyung Hee University, Seoul, Korea; 1 경희대학교 간호대학; 2 Department of Nursing, Ansan University, Ansan, Korea; 2 안산대학교 간호학과

**Keywords:** Health knowledge, Health literacy, Health promotion, Self concept, 건강지식, 건강정보 문해력, 건강증진, 자아존중감

## Abstract

**Purpose:**

The purpose of this study was to investigate the influence of eHealth literacy, reproductive health knowledge, and self-esteem on early adult women’s health-promoting behaviors (HPB). This study was based on Pender’s health promotion model as a theoretical underpinning.

**Methods:**

Early adult women aged 18 to 35 years (n=165) were recruited by posting advertisements on social network sites for a student club and a faith-based community in Ansan, Korea. Willing individuals were invited to participate in the online survey from June 1 to June 30, 2022. Standardized instruments were used to measure HPB, eHealth literacy, reproductive health knowledge, and self-esteem. General characteristics included income level, perceived subjective health, and internet usage time. The collected data were analyzed using the independent t-test, one-way analysis of variance, Pearson correlation coefficients, and multiple regression.

**Results:**

The mean age of the respondents was 21.97±3.87 years. The total HPB score was 120.69, corresponding to a moderate level; and the total scores for eHealth literacy (30.24), knowledge of reproductive health (23.04), and self-esteem (35.62) were higher than the midpoint. The model explained 53.3% of variance in HPB, and self-esteem (β=.48, *p*<.001) was the most influential factor. Other influential factors were, in descending order, higher economic level, higher subjective health status, greater eHealth literacy, and less internet use time (<2 hours/day).

**Conclusion:**

In order to promote the health of early adult women, counseling or programs that positively improve self-esteem appear promising, and eHealth literacy should be considered as a way to promote HPB using information technology.

## Introduction

보건복지부와 한국건강증진개발원에서 발표한 제5차 국민건강증진 종합계획(Health Plan 2030)에 여성의 생애주기별 맞춤형 건강정책이 포함되어 추진되고 있고[1], 지역사회 건강증진사업에서 여성의 건강증진 프로그램을 계획하는 등 건강진단 및 건강서비스 사각지대 해소와 건강증진 교육 내실화를 세부과제로 실천하고 있다[1,2]. 질병관리청에서는 여성의 생애주기뿐 아니라 사회·경제적 수준을 고려한 건강상태와 이슈를 제시하고 있고, 젊은 여성의 영양 섭취 부족이 심각한데, 이는 향후 여성의 건강과 가임력에 영향을 미쳐 저출산 등의 인구·사회학적 문제로 연결될 것이라고 하였다[2]. 18–25세는 성인기 진입 시기로 흡연, 음주, 약물 남용, 열악한 식습관 및 신체 활동 부족과 같은 건강에 해롭고 위험한 다양한 건강 행동을 하는 경향이 있는데, 이러한 행동 경향은 젊은 인구의 질병 위험 증가로 이어질 수 있다[3].

최근 연구동향과 연관된 선행연구를 살펴보면, 대학생의 건강증진행위(health promotion behavior) 관련 연구[4-6]에서 주관적 건강상태가 건강행위에 영향을 미치고, e헬스 문해력이 높을수록 건강행위 수준이 높다고 보고하였다. 여성의 건강행동과 여성건강증진 연구[6]에서는, 생애주기상 성인초기 여성들이 신체활동과 영양 균형의 중요성을 알고 있으면서도 건강행동으로 이어지지 못하며, 이를 실천하는 데 한계가 있다고 보고하였다.

우리나라 인터넷 이용실태를 살펴보면 대학생 포함 20대의 인터넷 이용률이 99.9%에 이른다[7]. 이러한 인터넷 사용자 수의 급격한 증가에 따라, 온라인 상에서 건강정보를 얻기 위해 정보통신기술을 이용하는 electronic health (eHealth, e헬스) 개념이 도입되었다[8]. 세계보건기구(World Health Organization)에서는 e헬스를 ‘건강을 위한 정보통신기술(information and communications technology)의 사용’으로 정의하였으며, e헬스 및 모바일 건강(mobile health) 애플리케이션이 취약한 지역사회 구성원의 의료 서비스 접근율을 개선할 수 있는 기회와 솔루션을 제공하기 때문에 e헬스는 보편적인 건강 보장을 달성하는 데 중요한 도구라고 발표하였다[7]. e헬스 문해력(eHealth literacy)은 온라인의 건강정보를 탐색, 선별, 이해, 평가할 수 있는 능력으로, 적절한 건강행위를 하도록 하는 요인으로 알려져 있다[9,10]. 이러한 e헬스 문해력은 투약, 운동, 식이관리 등의 행위를 상승시키는 것으로 조사되었으며[11], 보건의료 전공 학생들을 중심으로 한 연구[5,11]에서도 e헬스 문해력이 대학생의 건강증진행위를 향상시킬 수 있다고 보고한 바, 온라인 건강정보를 조사하는 것은 건강증진행위에서 비롯된 긍정적 태도라고 생각된다. 특히 성인초기 여성은 생식건강지식을 병원이나 전문 의료진에게서 얻기보다 대부분 친구나 인터넷, 텔레비전 등의 대중 전달매체를 통해 얻는 것으로 보고되고 있는데[12], 성인초기 여성의 e헬스 문해력이 건강증진행위에 긍정적인 영향을 미친다면 이와 관련된 변수를 확인하는 연구가 필요하리라 본다.

국내 대학생을 대상으로 조사한 연구[13]에서는 건강지각과 건강지식을 독립변수로 한 모형에서 건강증진행위 영향요인이 건강지식이라고 제시하면서 건강증진행위는 성별에 좌우된다고 하였다. 대학생들은 건강증진과 관련하여 직업 선택과 사회화, 결혼 및 부모됨 등의 성인중기를 위한 중요한 발달 과업을 성공적으로 이룰 수 있도록 건강증진행위를 해야 하는데[13], 특히 여성 건강정책은 저출산, 고령화 사회에 대응하기 위한 출산 장려정책과 모자보건정책 등에 집중되어[14] 임신과 출산 과업을 앞두고 있는 35세 이하의 성인초기 여성의 생식건강을 위한 적절한 실천의 필요성이 강조되고 있다. 생식건강이란 생식기관, 생식기능 및 생식과정과 관련된 건강뿐만 아니라 성생활, 자녀 출산의 결정권 및 생식권리 등을 포함한 총체적인 의미를 뜻하며[15], 생식건강지식은 생식기계 질환, 임신 및 출산, 가족계획, 인공유산, 성병, 성 건강 문제 등 생식기 구조와 기능 관련 지식을 의미한다[2]. 이러한 선행연구를 토대로 성인초기 여성의 생식건강지식을 건강증진행위에 주요한 영향요인으로 고려하고, 우선 생식건강지식 정도를 확인한 후 맞춤형 건강증진행위를 도모할 수 있는 방안을 마련할 필요가 있다. 게다가 미취업 성인초기 대학생은 건강검진 사각지대에 놓여있어 성인병으로 진행될 수 있는 건강의 이상증상을 조기 발견하여 예방할 수 있어야 한다[1]. 최근 가임기 여성의 성 활동이 활발해지고 있으나 상대적으로 이에 대한 예방적 건강행동이 부족한 성인초기에서 건강증진행동은 건강 이슈로 되짚어 볼 필요가 있다[2].

성인초기 여성의 건강증진행위와 연관된 중요한 요인으로 자아존중감이 있다[6]. 스스로 자신을 가치 있다고 평가하는 마음을 갖는 것은 매우 중요하며, 이러한 긍정적 자아존중감이 건강증진행위를 도모하는 역할을 한다[6]. 자아존중감이 높은 경우 삶을 대하는 태도가 긍정적이어서 건강증진행위를 적절하게 실천한다[16]. Pender는 생활실천을 통한 건강증진에 초점을 두고, 건강증진행위 관련 변인 간의 상호작용을 규명할 수 있는 다차원적인 접근이 가능하다고 하였다[17]. 이러한 선행연구를 기반으로 하여, 본 연구에서 성인초기 여성의 건강증진을 도모하기 위해서 사회·경제적 요인과 생식건강지식 등을 확대 적용하여 건강증진행위 영향요인을 분석할 필요가 있다.

본 연구는 결혼, 임신과 출산, 육아 등의 중요한 발달과업을 성공적으로 완수해야 하는 중요한 시기인 성인초기 여성을 대상으로 건강증진행위에 미치는 영향요인을 알아보고, 이를 통해 성인초기 여성의 건강을 유지, 증진하기 위한 효율적인 교육중재 방안을 마련하며, 건강정책과 건강증진행위 프로그램을 개발하는 데에 유용한 기초 자료가 될 것이다.

본 연구의 구체적 목적은 다음과 같다. 첫째, 대상자의 건강증진행위, e헬스 문해력, 생식건강지식, 자아존중감 정도를 파악한다. 둘째, 대상자의 특성에 따른 건강증진행위, e헬스 문해력, 생식건강지식, 자아존중감의 차이를 파악한다. 셋째, 건강증진행위, e헬스 문해력, 생식건강지식, 자아존중감의 상관관계를 파악한다. 넷째, 대상자의 건강증진행위에 미치는 영향요인을 파악한다.

## Methods

Ethics statement: This study was approved by the Institutional Review Board of Ansan University (AN01-202106-HR-003-01) and obtaining informed consent was exempted because there was no sensitive information and the online survey was anonymously treated.

### 연구 설계

본 연구는 성인초기 여성을 대상으로 일반적 특성과 e헬스 문해력, 생식건강지식, 자아존중감, 건강증진행위를 확인하며 건강증진행위에 영향을 미치는 요인들을 파악하기 위한 상관성 조사연구이다. 연구의 기술은 STROBE 보고지침(https://www.strobe-statement.org/)에 따라 작성하였다.

### 연구 대상

본 연구의 대상자는 경기도 안산시에 거주하고 연구 참여에 동의한 성인초기 여성을 모집단으로 편의 표집하였다. 대상자 선정기준은 만 18–35세 이내 성인초기 여성으로 연구 목적을 이해하고 연구 참여에 동의한 자로서 설문 내용을 이해하고 응답할 수 있는 여성이다. 본 연구 대상자 수 산정을 위해 G*Power 3.1.9.7 프로그램을 이용하여 회귀분석을 하였다. 유의수준 (α) .05, 검정력(1-β) .95, 중간수준의 효과크기 0.15로 설정하고[18] 예측변수(연령, 종교, 경제적 수준, 인터넷 사용시간, 주관적 건강상태, e헬스 문해력, 생식건강지식, 자아존중감) 기준에 의해 산출한 결과 연구 대상자 수는 160명이었으며, 탈락률 10%를 감안하여 176명을 대상자 수로 설정하였다. 최종 연구 대상자는 불성실한 응답자 11명을 제외한 총 165명이다.

### 연구 도구

본 연구의 도구는 도구의 원개발자 또는 번안자에게 전자우편과 유선으로 도구 사용 승인을 받은 후 사용하였다.

#### 건강증진행위

건강증진행위는 Walker 등[18]이 개발한 건강증진생활 양식(Health Promoting Lifestyle Profile II) 도구를 Yun과 Kim [19]이 번안한 도구를 사용하였다. 본 도구는 총 50문항으로, 건강책임 9문항, 영적 성장 9문항, 신체활동 8문항, 영양 8문항, 대인관계 8문항, 스트레스 관리 8문항의 6개 영역으로 구성된다. 각 문항은 ‘전혀 그렇지 않다’ 1점부터 ‘항상 그렇다’ 4점까지의 Likert 4점 척도로 측정되며, 점수의 범위는 50–200점으로 점수가 높을수록 건강증진행위 수행 정도가 높음을 의미한다. 개발 당시 도구[18]의 신뢰도 Cronbach’s α=.92였고, 번안한 도구[19]의 신뢰도 Cronbach’s alpha=.91이었으며, 본 연구에서의 Cronbach’s α=.95였다.

#### e헬스 문해력

e헬스 문해력은 Norman과 Skinner [9]가 개발한 eHealth literacy scale을 Park 등[20]이 번안한 도구를 사용하였다. e헬스 문해력은 단일 영역의 도구로 총 8문항이고, ‘전혀 그렇지 않다’ 1점부터 ‘매우 그렇다’ 5점까지의 Likert 5점 척도로 측정되며, 점수의 범위는 8–40점으로 점수가 높을수록 e헬스 문해력 수준이 높은 것을 의미한다. e헬스 문해력의 원 도구[9]의 신뢰도 Cronbach’s α=.88이었고, Park 등[20]이 번안한 도구의 Cronbach’s α= .875이었으며, 본 연구에서의 Cronbach’s α=.94였다.

#### 생식건강지식

생식건강지식은 Park과 Choi [15]가 개발한 생식건강지식 척도를 Cho [2]가 수정 보완한 도구를 사용하였다. 총 34문항으로 생식기의 구조 및 기능 6문항, 임신 및 출산 11문항, 피임 및 성 매개 감염 12문항, 생식기 암 5문항으로 구성되어 있다. 정답을 체크하면 1점, 오답 및 ‘모르겠다’로 체크한 경우는 0점으로 총 점수는 0–34점이며, 점수가 높을수록 생식건강지식이 높음을 의미한다. Park과 Choi [15]의 개발 당시 신뢰도는 Kuder-Richardson formulas 20 (KR-20)=.79였고, Cho [2]의 수정한 도구는 KR-20=.88이었으며, 본 연구의 KR-20=.71이었다.

#### 자아존중감

자아존중감은 Rosenberg [21]의 자아존중감 척도(self-esteem scale)를 Jeon [22]이 한국어로 번안하여 표준화한 설문지를 Lee 등[23]이 문항수준 신뢰도와 타당도 검증을 한 10문항을 사용하였다. 긍정적 자아존중 5문항, 부정적 자아존중 5문항의 두 영역으로 ‘전혀 그렇지 않다’ 1점부터 ‘항상 그렇다’ 5점까지 Likert 5점 척도로 측정하며, 점수의 범위는 10–50점으로 점수가 높을수록 자아존중감의 정도가 높음을 의미한다. Lee 등[23]의 도구의 신뢰도는 Cronbach’s α=.75–.87이었고, 본 연구에서의 Cronbach’s α=.90이었다.

#### 대상자 특성

대상자 특성은 연령, 종교, 경제적 수준, 인터넷 사용시간, 주관적 건강상태 총 5문항으로 구성하였다. 경제적 수준은 상, 중, 하로 구분하였고, 인터넷 사용시간은 2시간 미만, 2–3시간, 4시간 이상으로 구분하였으며, 주관적 건강상태는 전혀 건강하지 않음, 건강하지 않음, 건강함, 매우 건강함으로 구분하였다.

### 자료 수집

자료 수집 기간은 2022년 6월 1일부터 30일까지였으며, coronavirus disease 2019 (COVID-19) 상황을 고려하여 경기도 안산시 안산대학의 대학교회 청년부와 일반동아리 회원을 대상으로 모집 공고문으로 홍보하여 관심자가 인터넷 링크를 통해 접속하도록 하였다. 온라인 설문지 첫 화면에 연구 대상자 선정 조건을 제시하여 부합 여부를 확인하고, 자료 수집 시 대상자가 설문을 시작하기 전 첫 화면에 연구내용에 대한 설명서와 연구 대상자 제외 기준 등에 관한 안내문을 온라인으로 제공하였다. 설문조사 소요 시간은 15분 정도였고, 연구 참여자에게는 5,000원 상당의 커피 모바일 쿠폰을 제공하였다.

### 자료 분석 방법

수집된 자료 분석은 IBM SPSS ver. 23.0 (IBM Corp., Armonk, NY, USA)을 이용하였다. 대상자의 특성, 건강증진행위, e헬스 문해력, 생식건강지식, 자아존중감 정도는 빈도, 백분율, 평균, 표준편차 등 기술통계로 분석하였고, 대상자의 특성에 따른 건강증진행위, e헬스 문해력, 생식건강지식, 자아존중감의 차이는 독립 t검정, 일원분산분석, 사후 검정은 Scheffé test로 분석하였다. 건강증진행위, e헬스 문해력, 생식건강지식, 자아존중감의 상관관계를 파악하기 위하여 Pearson 상관계수로 분석하였고, 대상자의 특성, e헬스 문해력, 생식건강지식, 자아존중감이 건강증진행위에 미치는 영향을 파악하기 위하여 다중회귀분석(multiple regression analysis)을 이용하였다.

## Results

### 대상자의 특성에 따른 건강증진행위, e헬스 문해력, 자아존중감 및 생식건강지식

연구 대상자의 평균 나이는 21.97±3.87세였고, 20세 이하가 78명(47.3%)이었다. 종교는 없음이 115명(69.7%)이었으며, 경제적 수준은 ‘중’인 경우가 124명(75.2%)이었고, 인터넷 사용시간은 4시간 이상이 83명(50.3%)으로 가장 많았으며, 주관적 건강상태는 건강한 편인 경우가 108명(65.5%)으로 가장 많았다(Table 1).

건강증진행위의 총점은 120.69±22.5점(평균 평점, 2.41점)으로 중등도 수준이었고, 대상자의 특성 중 종교가 있는 경우가 없는 경우보다 더 높았다(t=–2.36, *p*=.020). 경제적 수준이 ‘상’인 경우가 총점이 가장 높았으며(F=8.08, *p*<.001), 경제적 수준이 ‘하’, ‘중’인 집단과 ‘상’인 집단 간에 유의한 차이가 있는 것으로 나타났다(Table 1). 인터넷 사용시간이 2시간 미만인 경우가 가장 높았고(F=4.25, *p*=.016), 2시간 미만 집단과 4시간 이상 집단 간에 유의한 차이가 있는 것으로 나타났다. 주관적 건강상태는 매우 건강한 집단에서 가장 높았고(F=10.92, *p*<.001), ‘전혀 건강하지 않음’과 ‘건강하지 않음’ 집단 간과 ‘건강함’과 ‘매우 건강함’ 집단 간에 유의한 차이가 있는 것으로 나타났다.

e헬스 문해력의 총점은 30.24±5.75점(평균 평점, 3.78점)으로 중등도 이상의 수준이었으며, 대상자의 특성에 따른 통계적으로 유의한 차이는 없는 것으로 나타났다. 생식건강지식의 총점은 23.04±0.32점으로 중등도 이상의 수준이었고, 대상자의 연령에 따라 유의한 차이를 보이는 것으로 나타났으며, 연령이 26세 이상인 경우 생식건강지식이 가장 높은 것으로 나타났다(F=11.69, *p*<.001). 자아존중감의 총점은 35.62±4.71점으로 중등도 이상의 수준이었고, 대상자의 종교, 경제적 수준, 인터넷 사용시간과 주관적 건강상태에 따라 차이를 보였다. 종교가 있는 경우가 없는 경우보다 높았고(t=–2.15, *p*=.033), 경제적 수준이 상인 경우가 가장 높았으며(F=6.02, *p*=.003), 인터넷 사용시간이 2시간 미만인 경우(F=3.82, *p*=.024)와 주관적 건강상태가 매우 건강한 경우(F=12.93, *p*<.001)에서 자아존중감이 가장 높았다(Table 1).

### 건강증진행위와 e헬스 문해력, 생식건강지식, 자아존중감의 관계

대상자의 건강증진행위와 e헬스 문해력(r=.37, *p*<.001)은 약한 양의 상관관계, 건강증진행위와 자아존중감(r=.61, *p*<.001)은 중간 정도 양의 상관관계로 e헬스 문해력과 자아존중감 점수가 높을수록 건강증진행위 점수가 높게 나타났다. e헬스 문해력과 생식건강지식(r=.31, *p*<.001)은 약한 양의 상관관계, e헬스 문해력과 자아존중감(r=.28, *p*<.001)도 약한 양의 상관관계가 있는 것으로 나타났다(Table 2).

### 건강증진행위에 미치는 영향요인

대상자의 건강증진행위에 영향을 미치는 요인을 확인하기 위해 대상자 특성 중 건강증진행위에 유의한 차이를 보였던 대상자의 종교, 경제적 수준, 인터넷 사용시간, 주관적 건강상태, e헬스 문해력, 생식건강지식, 자아존중감을 통제변수로 투입하였다. 이중 종교, 경제적 수준, 인터넷 사용시간, 주관적 건강상태를 더미변수로 처리하여 투입하여 다중회귀분석을 한 결과, 자아존중감(β=.48, *p*<.001)이 건강증진행위에 가장 영향력이 높았고, 다음은 경제적 수준이 하인 경우(β=–.42, *p*=.003)와 중인 경우(β=–.39, *p*=.004), 주관적 건강상태가 건강하지 않음의 경우(β=.31, *p*=.018), 인터넷 건강정보 문해력 수준이 높은 경우(β=.18, *p*=.005), 인터넷 사용시간이 2시간 미만인 경우(β=.14, *p*=.026) 순이었다. 회귀분석에서 이 독립변수의 선형성 및 등분산성을 검정하기 위해 산점도를 확인한 결과, 잔차의 분포가 0을 중심으로 균등하게 흩어져 있으므로 가정을 충족하였다. 그리고 회귀 표준화 잔차의 정규 P-P 도표를 이용하여 오차의 정규분포를 확인한 결과 45° 직선에 근접해 있으므로 오차는 정규분포를 이룬다. 오차의 독립성을 검증하기 위해 Durbin-Watson 통계량을 확인한 결과 2.364로 2에 가까워, 모형의 오차항 간에 자기상관성은 없는 것으로 나타났다. 공차한계(tolerance)는 0.17–0.96으로 0.1 이상이었으며, 분산팽창지수는 1.05–5.98로 10 미만이므로 독립변수들 간에 다중공선성의 문제가 없음을 확인하였다. 회귀식의 적합도를 검정한 결과 회귀식이 유의하였고(F=15.90, p <.001), 설명력은 53.3%였다(Table 3).

## Discussion

본 연구에서는 성인초기 여성의 건강정보 문해력, 생식건강지식, 자아존중감, 건강증진행위 정도를 파악하고, 이에 미치는 영향요인을 중심으로 이에 미치는 영향요인을 중심으로 논의하면 다음과 같다.

대상자의 건강증진행위 정도는 동일한 측정 도구를 이용한 선행연구에서 총점 대신 평균 평점을 제시한 것과 비교하면, 본 연구는 2.41로 간호 대학생의 건강정보 추구 행동이 건강증진행위 실천에 미치는 영향 연구[12]에서 제시한 2.21점과 여대생의 건강증진행위 점수가 2.30점으로 보고한 연구[24]보다 높게 나타났다. 건강증진행위는 경제적 수준이 ‘상’인 집단에서 수행 정도가 가장 높았는데, Lee와 Nam [5]의 연구에서는 대학생의 경제적 수준이 ‘상’인 집단에서 건강증진행위 수행 정도가 높다고 제시하였으나 유의미한 차이는 없는 것으로 나타났다. 인터넷 사용시간은 ‘2시간 미만’의 경우에서 건강증진행위 정도가 가장 높게 나타났는데, Lee와 Nam [5]의 연구에서는 ‘2–3시간’ 사용 집단에서 높게 나타났다. 한편 본 연구와 상반된 결과를 보고한 Shin [25]에 의하면, 인터넷 사용시간이 6시간 이상인 경우에 건강증진행위 정도가 가장 높게 나타났다. 통상적으로는 인터넷 사용시간이 많으면 사람들이 원하는 다양한 정보를 검색하여 건강증진행위가 향상할 것이라고 생각할 수 있으나, 본 연구의 결과로 보면 건강한 성인초기 대상자의 인터넷 장시간 사용과 건강증진행위 실천과는 대체로 관련이 없다고 여겨진다. 주관적 건강상태는 ‘매우 건강함’의 경우에 건강증진행위 수행 정도가 가장 높았으며, 대학생의 건강증진행위 영향요인 연구[6,24]와 청장년기의 건강증진행위에 미치는 영향요인 연구[26]에서도 본 연구 결과와 유사한 결과를 제시한 바, 주관적 건강은 사회·문화적, 신체적, 환경적 요소의 영향을 받을 수 있고, 개인적으로 인지된 신체·정신적 증상은 건강증진행위에 영향을 미칠 수 있다는 것을 알 수 있었다[4].

e헬스 문해력 수준은 동일 도구를 사용했으나 총점 대신 평균 평점을 보고한 선행연구와 비교 시 3.78로 높았다. 즉, Park과 Kim [27]의 연구에서는 간호대생은 3.67점, 비간호대생은 3.17점으로 보고하였고, 여대생의 e헬스 문해력이 3.59점이라고 제시한 연구[24]와 의학 및 건강과학 대학생의 e헬스 문해력 수준이 24.47점이라고 보고한 연구[28]보다도 본 연구에서 높은 수준을 나타낸 바, 최근 계속해서 인터넷 활용이 증가하고 정보통신 기기가 상용화되면서 e헬스 문해력 수준이 증가하고 있는 것으로 생각된다. 한편, 본 연구의 대상자 특성에 따른 e헬스 문해력은 통계적으로 유의한 차이를 보이지 않았는데, Lee와 Nam [5]의 연구에서는 건강 관심도, 건강관리 시간에 따른 유의한 차이를 보였다. 이러한 특성은 e헬스 문해력과 연관성이 있을 것으로 보여 추후 연구에 포함하여 분석해야 할 것으로 보인다.

생식건강지식은 평균 23.04점(34점 만점)으로 여대생의 생식건강에 대한 지식수준을 24.35점(35점 만점)으로 보고한 연구 결과[29]와 유사하였고, 대학생의 생활습관과 생식건강과의 관계 연구[30]에서는 여학생의 생식건강지식을 19.72점(35점 만점)으로 보고하여 본 연구 결과보다 낮게 나타났다. 이러한 결과는 COVID-19 시대 감염 예방수칙과 관련한 지식과 실천에 e헬스 문해력이 적극적으로 활용되면서 연구에서 생식건강지식 점수가 높게 나타났기 때문일 수 있다. Cho [2]의 성인초기 여성을 대상으로 한 연구에서는 생식건강증진 프로그램 중재 전 24.73점(34점 만점)에서 중재 후 28.03점으로 생식건강지식 점수가 향상된 것으로 보고한 바, 생식건강증진 프로그램 중재를 통해 생식건강지식을 고양할 수 있다 하겠다. 또한 성인초기 여성에게 생식건강 관련 문제, 증상 및 관리 등을 위한 생식건강 교육을 통해 지식수준을 높일 수 있다고 한 연구는[31] 생식건강 교육프로그램 개발 및 적용의 필요성을 시사한다.

자아존중감은 가능점수 10–50점 중 35.62점이었으며, 대학생의 자아존중감 연구[32]에서 100점 만점을 기준으로 보고한 수준(여학생, 86.91점; 남학생, 89.05점)보다 낮게 나타났는데, 이러한 결과는 터키와 우리 나라 대학생 간의 개인 사회 발달 특성과 연관성이 있을 것으로 생각한다. 대상자의 경제적 수준별 자아존중감은 경제적 수준이 ‘상’인 집단에서 자아존중감 점수가 가장 높았고, 대상자의 소득수준이 높을수록 자아존중감이 더 많이 증가하는 것으로 나타났는데, 사람들은 삶에서 일정한 소득이 있을 때 능동적 역할을 할 수 있으며 유능하고 가치있다고 느끼므로[32], 경제적 수준과 자아존중감 사이에는 관계가 있다고 기대할 수 있다. 주관적 건강상태별 자아존중감은 ‘매우 건강함’을 지각하는 경우에서 더 높게 나타났다. Lee와 Song [33]은 성인초기 건강행동과 관련한 중요한 개인 요인이 자아존중감이라고 제시한 바, 자아존중감이 높은 집단에서는 자신의 건강상태를 지각하여 주도적으로 스스로 건강을 관리하는 긍정적인 선택적 건강행위를 할 수 있을 것이다[6].

성인초기 여성의 건강증진행위에 영향을 미치는 요인에 대한 회귀분석에서는 건강증진행위에 대한 자아존중감의 영향이 가장 큰 것으로 나타났고, 경제적 수준, 주관적 건강상태, e헬스 문해력, 인터넷 사용시간 순으로 나타났다. 청소년기와 간호대학생의 건강증진행위에 대한 선행연구[6,16]에서도 건강증진행위에 대한 자아존중감이 큰 영향요인으로 나타나 본 연구 결과와 일치하였다. 자아존중감이 높은 군에서 자기효능감, 스트레스, 피로 수준을 건강증진행위의 영향요인으로 보고한 연구[6]에 의하면, 자아존중감이 높으면 스트레스가 낮고, 친구들과 협력관계 유지로 대인관계가 원활하여 삶에 대한 긍정적 태도를 가지고 건강행위를 한다. 반면 자아존중감이 낮으면 스트레스 정도가 높아 건강행위 불이행과 더불어 건강문제를 겪는다[6]. Lee와 Song [33]에 의하면, 건강증진행위와 관련된 주요 요인으로 자아존중감은 인간이 행복감을 느끼는 데 필요한 요건으로, 자아존중감이 높을수록 모든 일에 적극적이어서 건강증진행위의 실천 정도가 높아지게 된다[34]. 따라서 자신의 건강에 대해 관심이 많고 자신을 존중하는 사람은 정확한 정보를 탐색하고 이해하고자 하므로, 성인초기 여성의 건강증진행위 수행을 촉진하기 위해서는 자아존중감을 높이는 방안을 마련함과 동시에 이를 강화할 수 있는 중재 전략이 필요함을 시사한다.

본 연구에서 경제적 수준이 건강증진행위에 영향을 미치는 요인으로 나타났으며, 경제적 수준은 ‘하’와 ‘중’인 경우보다 ‘상’인 경우 건강증진행위 정도가 높고, 인터넷 사용시간이 ‘2시간 미만’인 경우가 ‘4시간 이상’인 경우보다 건강증진행위 정도가 더 높았다. 반면, 경제적 수준과 건강증진행위 간에 유의한 차이가 없는 것으로 보고한 연구[5,19]도 있다. 따라서 경제적 수준이 성인초기 여성의 건강증진행위에 영향을 미치는 이유를 단정짓기에는 무리가 있으나, 경제 상태에 따라 건강증진행위에 유의한 차이를 나타낸 연구[26]를 바탕으로 생각해 볼 수 있는 것은 경제수준이 높은 경우 보건, 병원 시설을 방문할 가능성이 높으므로 이러한 경제적 요건이 예방적 건강증진행위에 긍정적 영향을 미쳤을 것이라는 추론이다. 인터넷 사용시간도 건강증진행위에 영향을 미치는 요인으로 나타났는데, Hwang과 Oh [35]의 연구에서도 건강 관련 정보를 온라인에서 검색하는 데 사용한 주당 시간이 건강증진행동의 영향요인으로 나타났다. 한편, Lee와 Nam [5]과 Shin [25]의 연구에서는 사회·경제적 수준과 인터넷 사용시간이 건강행위에 영향을 미치지 않는 것으로 보고하였다. 이러한 결과는, 통상적으로 인터넷 사용시간이 많으면 다양한 정보를 검색하는 시간이 증가할 것으로 생각하지만 실제적으로 많은 정보 중에서 정확한 건강 정보를 찾고 해석하는 능력과는 무관할 수도 있음을 시사한다.

e헬스 문해력도 건강증진행위에 영향을 미치는 요인으로 확인되었는데, 이는 e헬스 문해력이 건강증진행위에 통계적으로 유의미한 영향을 미친다는 선행연구들[20,33]을 지지한다. 건강증진행위에 미치는 영향요인을 주관적 건강상태와 e헬스 문해력 순으로 보고한 연구[36]에 의하면, 주관적 건강상태 ‘좋음’의 경우 인터넷 건강정보를 얻으려는 경향이 더 강하다고 하였다. 이러한 결과를 통해 e헬스 문해력 수준을 높이면 자기 건강을 관리하기 위한 건강증진행위를 적극적으로 실천할 것으로 기대해 본다. 따라서 e헬스 문해력과 건강증진행위 간에 상관성과 영향력이 있으므로, 정확한 건강정보를 얻기 위해 정보기술을 활용할 수 있는 기초를 배우도록 할 필요가 있다. 이러한 정보기술 활용능력은 실천 정도를 높일 수 있는 방안임을 시사해준다. 국가 차원에서도 국민들의 e헬스 문해력 수준을 높여 개인의 건강관리를 위해 여성을 위한 교육 기회 확대 및 권한을 부여하는 동시에 인구 증가 지원을 위해 꾸준한 노력을 하고 있는 것을 감안할 때[5], 정보기술을 활용하여 건강증진행위를 향상할 수 있도록 홍보하기 위해 e헬스 문해력을 고려해야 할 것이다. 따라서 성인초기 여성의 건강증진행위 정도를 높이고 실천을 촉진하는 데 e헬스 문해력을 통한 중재 전략을 세우는 것이 중요하다.

한편, 생식건강지식은 건강증진행위에 영향을 미치지 않는 것으로 나타났는데, 생식건강행위를 생식건강에 영향을 주는 생활습관으로 보고 흡연, 음주, 약물 남용, 다이어트, 규칙적 운동과 식사, 철분 보충, 유방암과 자궁암 자가검진의 행위에 대해 측정한 연구[29]에 의하면 생식건강지식 수준이 높을수록 생식건강 관련 습관이 더 형성될 수 있다고 보고하였다. 여대생의 여성건강지식과 건강행위 실천의 변화 연구[37]에서는 여성건강 관련 교육 전후 대상자의 건강행위 실천 정도가 유의하게 증가하였는데, 여성건강지식은 본 연구의 생식건강지식과 유사하게 생식기의 구조 및 기능, 임신과 분만 및 산후 관리, 피임 관리, 인공 임신중절 예방, 성접촉 성질환 예방, 생식기 감염질환 관리, 여성 생식기 암 관리의 내용을 포함하고 있다. 여대생의 생식건강증진행위는 안전한 성행위, 성행위 책임감, 생식기 건강관리, 성병 예방, 생식기 위생관리를 측정한 연구[38]에서 성지식이 생식건강증진행위의 영향요인으로 나타난 바, 본 연구에서 건강증진행위 영향요인으로 생식건강지식을 확인했다는 점에서 연구의 한계가 있었다.

본 연구의 제한점은 건강증진행위 영향요인을 예측함으로써 성인초기 여성의 실천을 도모할 수 있는 간호중재의 전략을 제시하였지만 경기도 안산에서 편의 추출하였으므로 연구 결과를 일반화하는 데 어려움이 있다. 향후 연구를 위한 제언으로, 건강에 대한 인식[3]과 건강관심도[5]가 건강증진행위에 영향을 미치는 변수임을 규명할 필요성을 제시하며, 성인초기 여성의 건강증진행위 실천을 강화하기 위해서 e헬스 문해력을 활용하도록 홍보하고, 자아존중감을 높이는 전략이 포함된 교육 프로그램을 개발할 것을 제언한다.

본 연구를 통해 성인초기 여성의 건강증진행위 영향요인으로 확인된 자아존중감, 경제적 수준, 주관적 건강상태, e헬스 문해력은 간호중재를 제공할 때 접근의 방향을 제시할 수 있는데, 즉 e헬스 문해력 수준을 높여 건강정보를 활용하는 방법을 교육하여 건강관리 실천 효과를 기대할 수 있다. 또한 경제적 수준을 고려하여 건강증진을 위한 건강서비스를 활용하고, 주관적 건강상태에 따른 맞춤형 보건 교육 정책과 교육 프로그램을 실시하여 성인초기 여성들의 건강증진행위 실천을 도모할 수 있다고 생각한다.

## Figures and Tables

**Table 1.** Differences in HPB, eHealth literacy, RHK, and self-esteem according to participants’ characteristics (N=165)

Variable	Categories	n (%)	HPB		eHealth literacy		RHK		Self-esteem	
			Mean±SD	Range or t or F (*p*)	Mean±SD	Range or t or F (*p*)	Mean±SD	Range or t or F (p)	Mean±SD	Range or t or F (*p*)
			120.69±22.54	76–200	30.24±5.75	8–40	23.04±.32	10–32	35.62±4.71	22–46
Age (year)	18–20^a^	78 (47.3)	122.00±20.25	1.43 (.242)	29.76±6.13 30.83±5.81	.61 (.546)	21.82±4.15	11.69 (<.001)	35.68±4.04	2.06 (.131)
	21–25^b^	64 (38.8)	121.75±25.16		30..26±4.07		23.37±.3.57	(a<b<c)^†^	36.17±5.17	
	26–35^c^	23 (13.9)	113.35±21.78				26.72±4.15		33.87±5.23	
Religion	None	115 (69.7)	118.00±21.02	–2.36 (.020)	30.22±5.53	–.084 (.933)	23.11±.3.94	.36 (.720)	35.10±4.65	–2.15 (.033)
	Yes	50 (30.3)	126.88±24.81		30.30±6.29		22.86±4.61		36.80±4.66	
Economic level	Lower^a^	35 (21.2)	111.00±18.29	8.08 (<.001)	28.57±5.37	2.87 (.059)	22.86±4.17	.97 (.326)	33.69±4.96	6.02 (.003)
	Middle^b^	124 (75.2)	122.19±21.86	(a<b<c)^†^	30.54±5.85		23.01±4.19		35.96±4.49	(a<b,c)^†^
	Upper^c^	6 (3.6)	148.50±33.58		33.83±3.25		24.67±3.20		39.83±3.64	
Internet use time (hour/day)	<2^a^	14 (8.5)	134.39±32.76	4.25 (.016)	30.86±6.61	.10 (.907)	23.50±3.82	.14 (.872)	37.07±5.27	3.82 (.024)
	2–3^b^	68 (41.2)	122.68±22.43	(a>c)^†^	30.26±4.72		23.10±3.87		36.51±4.67	(a>c)^†^
	≥4^c^	83 (50.3)	116.77±19.60		30.12±6.41		22.90±4.44		34.64±4.49	
Subjective health status	Not healthy at all^a^	6 (3.6)	93.33±8.33	10.92 (<.001)	26.00±0.89	2.21 (.089)	24.00±4.14	.32 (.814)	27.83±4.36	12.93 (<.001)
	Not healthy^b^	43 (26.1)	111.35±22.73	(a,b<c,d)^†^	39.36±0.76		22.91±3.75		33.84±4.46	(a,b<c,d)^†^
	Healthy^c^	108 (65.5)	124.18±20.05		30.64±0.68		22.95±4.35		36.46±4.26	
	Very healthy^d^	8 (4.8)	144.50±24.43		32.88±0.71		24.13±4.15		39.63±2.88	

HPB: Health-promoting behaviors; RHK: reproductive health knowledge.

† Scheffé test.

**Table 2.** Relationships among HPB, eHealth literacy, RHK, and self-esteem (N=165)

Variable	r (*p*)		
	HPB	eHealth literacy	RHK
HPB	1		
eHealth literacy	.37 (<.001)	1	
RHK	.04 (.653)	.31 (<.001)	1
Self-esteem	.61 (<.001)	.28 (<.001)	.10 (.191)

HPB: Health-promoting behaviors; RHK: reproductive health knowledge.

**Table 3.** Factors influencing health-promoting behaviors (N=165)

Variable	ß	SE	β	t	*p*
Religion^†^	4.23	2.76	.09	1.53	.128
Economic level, lower^†^	–22.81	7.43	–.42	–3.07	.003
Economic level, middle^†^	–20.46	7.00	–.39	–2.92	.004
Internet use time, <2 hour/day^†^	10.89	4.85	.14	2.25	.026
Subjective health status, not healthy^†^	–15.79	6.62	–.31	–2.39	.018
eHealth literacy	0.70	0.24	.18	2.87	.005
Reproductive health knowledge	–0.46	0.32	–.09	–1.45	.150
Self-esteem	1.73	0.25	.48	6.88	<.001
	Adjusted R^2^=.533, F(*p*)=15.90, *p* <.001				

† The reference groups were religion (none), economic level (upper), internet use time (≥ 4 hours/day), and subjective health status (very healthy).
